# Moonstruck Primates: Owl Monkeys (*Aotus*) Need Moonlight for Nocturnal Activity in Their Natural Environment

**DOI:** 10.1371/journal.pone.0012572

**Published:** 2010-09-03

**Authors:** Eduardo Fernández-Duque, Horacio de la Iglesia, Hans G. Erkert

**Affiliations:** 1 Department of Anthropology, University of Pennsylvania, Philadelphia, Pennsylvania, United States of America; 2 CECOAL-Conicet, Corrientes, Argentina; 3 Department of Biology, University of Washington, Seattle, Washington, United States of America; 4 Institute for Zoology, University of Tübingen, Tübingen, Germany; Texas A&M University, United States of America

## Abstract

Primates show activity patterns ranging from nocturnality to diurnality, with a few species showing activity both during day and night. Among anthropoids (monkeys, apes and humans), nocturnality is only present in the Central and South American owl monkey genus *Aotus*. Unlike other tropical *Aotus* species, the Azara's owl monkeys (*A. azarai*) of the subtropics have switched their activity pattern from strict nocturnality to one that also includes regular diurnal activity. Harsher climate, food availability, and the lack of predators or diurnal competitors, have all been proposed as factors favoring evolutionary switches in primate activity patterns. However, the observational nature of most field studies has limited an understanding of the mechanisms responsible for this switch in activity patterns. The goal of our study was to evaluate the hypothesis that masking, namely the stimulatory and/or inhibitory/disinhibitory effects of environmental factors on synchronized circadian locomotor activity, is a key determinant of the unusual activity pattern of Azara's owl monkeys. We use continuous long-term (6–18 months) 5-min-binned activity records obtained with actimeter collars fitted to wild owl monkeys (n = 10 individuals) to show that this different pattern results from strong masking of activity by the inhibiting and enhancing effects of ambient luminance and temperature. Conclusive evidence for the direct masking effect of light is provided by data showing that locomotor activity was almost completely inhibited when moonlight was shadowed during three lunar eclipses. Temperature also negatively masked locomotor activity, and this masking was manifested even under optimal light conditions. Our results highlight the importance of the masking of circadian rhythmicity as a determinant of nocturnality in wild owl monkeys and suggest that the stimulatory effects of dim light in nocturnal primates may have been selected as an adaptive response to moonlight. Furthermore, our data indicate that changes in sensitivity to specific environmental stimuli may have been an essential key for evolutionary switches between diurnal and nocturnal habits in primates.

## Introduction

Primates show activity patterns that range from nocturnality to diurnality, with a few species showing activity both during the day and night [1]–[4]. Among anthropoids (monkeys, apes and humans), nocturnality is only present in the Central and South American owl monkeys (*Aotus spp*), relatively small (approx. 1 kg), arboreal, socially monogamous primates that range from Panamá to Argentina [5], [6]. Observational studies have shown that most species in the genus are nocturnal [5], but the Azara's owl monkeys (*A. azarai*) of the subtropical Gran Chaco of Argentina and Paraguay have switched their activity pattern from strict nocturnality to one that also includes regular diurnal activity [7]. Harsher climate, food availability, and the lack of predators or diurnal competitors, have all been proposed as possible ultimate environmental factors favoring evolutionary switches in the activity patterns of primates [1], [6], [8]–[18], and other mammals [19]–[22]. However, the observational nature of field studies, the results of which frequently depend on the observers' activity rhythms and are biased by impaired vision during dark moonless nights, has generally limited our understanding of the proximate mechanisms responsible for the change in activity patterns of cathemeral species.

The *A. azarai* population of northern Argentina [23], [24] offers a unique opportunity to identify the environmental and biological factors that influence the distribution of activity across the 24-h day, which results in this species-specific cathemeral activity pattern [25]. In contrast to other owl monkeys, the population is located barely outside the tropics (58° 11′ W, 25° 58′S) where annual fluctuations in photoperiod and temperature generate appropriate conditions for analysing the effects of each environmental factor. The goal of our study was to evaluate the hypothesis that masking, namely the stimulatory and inhibitory effects of environmental factors on synchronized circadian locomotor activity [26], [27], is a key proximate determinant of the unusual activity pattern of Azara's owl monkeys.

The daily distribution of activity results from an interplay between two control mechanisms: an endogenous (i.e. circadian) timing system synchronized (entrained) to the light-dark (LD) cycle, and the ‘masking’ of the resulting circadian activity pattern by inhibiting or enhancing direct effects of light and other environmental factors [28]. The goal of the present study was to non-invasively establish how these two regulatory mechanisms may play out as determinants of the temporal distribution of locomotor activity in owl monkeys living in their natural habitat. We evaluated whether the species-specific pattern of activity fits a model in which circadian nocturnal locomotor activity is negatively masked (inhibited) by the low luminance levels during new-moon nights and cold environmental temperatures, and positively masked (disinhibited or enhanced) by higher luminance levels during moonlit nights. Additionally, we tested the prediction that the high activity levels expected during full-moon nights would be inhibited during three total lunar eclipses. We captured 10 *A. azarai* individuals in the gallery forests along the Guaycolec River in the Province of Formosa, Argentina [29] and fitted them with actimeter collars (Actiwatch® AW4 accelerometer/data logger devices), programmed to record and store activity in 5-min intervals, for periods that ranged between 6 and 18 months. All animals were recaptured 3–6 months later to retrieve the collars and/or re-fit them with a newly programmed one. Our data indicate that although the circadian system of *A. azarai* is programmed for a nocturnal activity pattern, masking by environmental light and temperature is a key determinant for the expression of nocturnality.

## Results and Discussion

### Nocturnal and diurnal activity in owl monkeys

**Figure 1:**
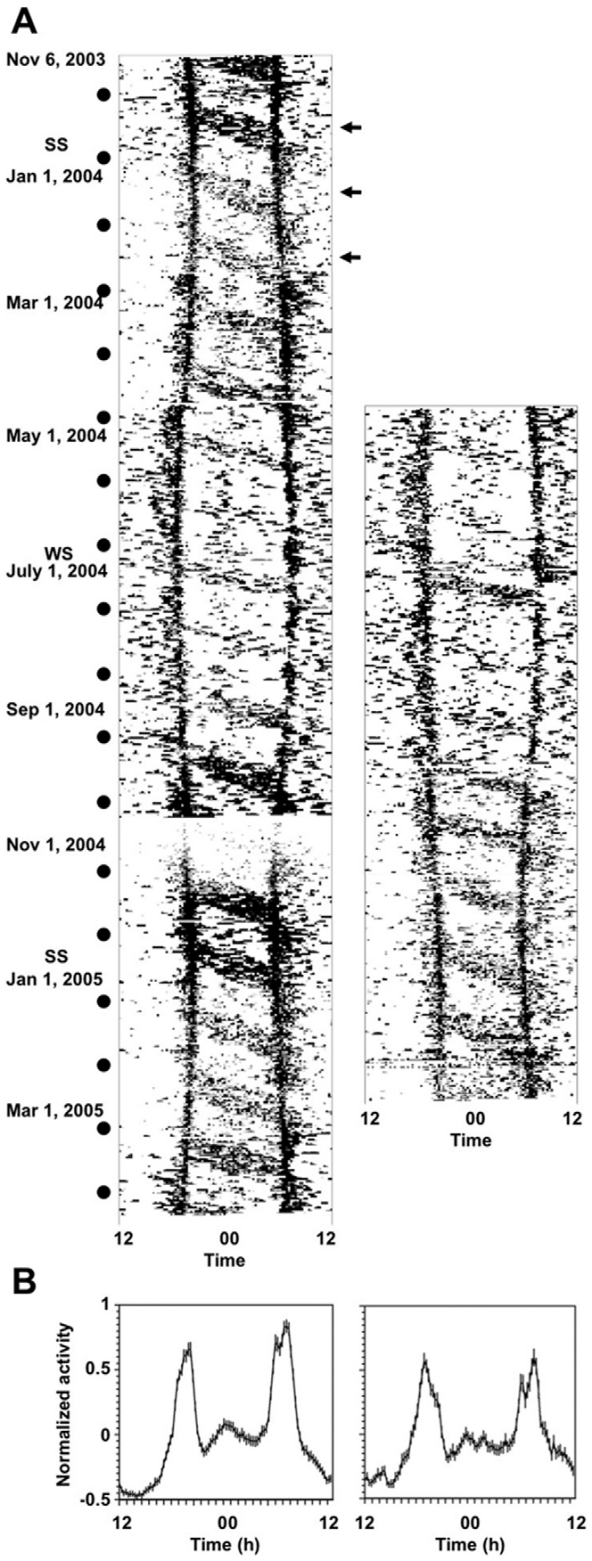
Locomotor activity patterns of two *A. azarai* males free-ranging in their natural environment. **A.** Double plot of original activity recordings. Days are stacked vertically and black bars indicate the average locomotor activity during 15 min throughout each 24-h period. Black circles represent new-moon days. SS, summer solstice, WS, winter solstice. Arrows highlight representative mornings of lower activity following full-moon nights. **B.** Mean wave profiles of the daily activity of the same animals shown in (a). Each point represents the average locomotor activity taken for each 15-min interval throughout the recordings shown in (A). Bars represent standard errors of the mean.

The activity of owl monkeys was predominantly restricted to dawn and dusk, and had a nocturnal component that was clearly associated with the lunar cycle (Figure 1A, B). The activity pattern of all 10 individuals, illustrated in Figure 1A by two representative individuals, was in solid agreement with data reported previously under laboratory conditions for other owl monkey species [30]–[31], and with a shorter-term study of *A. azarai* in the wild [7]. Nocturnal activity was more consolidated during the relatively warmer months of September to March than during the colder months of April to August, when temperatures in the Argentinean Chaco regularly fall below 10°C [24], [32]. Throughout the year, nocturnal activity (21:00–06:00 h) was higher during full-moon nights (51.6±1.1% of daily total activity) than during new-moon ones (25.9±1.0%; Wilcoxon signed-ranks test, two-tailed, p = 0.005, z = −2.803, n = 10 individuals) and these peaks of nocturnal activity were consistently followed by mornings of low activity (Fig. 1A). Conversely, new-moon nights were usually followed by mornings of higher diurnal activity (06:00–09:00 h, 26.7±0.7% of daily total activity) than mornings following full-moon nights (14.4±0.7%; Wilcoxon signed-ranks test, two-tailed, p = 0.005, z = −2.803, n = 10 individuals). The daily profile of activity, irrespective of season and lunar month, showed prominent dawn and dusk peaks with more predominant activity during the night than during the day (Figure 1B). A similar pattern was observed in all 10 animals studied.

Our analysis of locomotor activity in *A. azarai*, recorded remotely, non-invasively and with high time-resolution throughout several months is consistent with several observational studies in nocturnal primates that have found higher activity during full-moon nights [4], [8], [11], as well as with a short study of *A. azarai* and one of red-fronted lemurs (*Eulemur fulvus)* in which activity was recorded with actimeter collars [16], [33].

### Seasonal changes and a dual oscillator model

Circadian clocks have a period that is close, but not equal, to 24 h. Therefore they need to be entrained by 24-h environmental cycles. The light-dark (LD) cycle is the most pervasive and precise entraining agent and a circadian rhythm is entrained when it bears a constant phase relationship to it. According to a so-called discrete (also known as non-parametric) model of entrainment, this constant phase relationship between the rhythm and the environmental cycle can be achieved by daily shifts in the phase of the circadian clock that drives the rhythm [34], [35]. These daily phase corrections would compensate the difference between the circadian clock period and the environmental cycle. Previous laboratory studies of the Colombian owl monkey, *A. lemurinus griseimembra*, indicated that entrainment to LD cycles was in line with the discrete model of entrainment [36]. Furthermore, evidence from nocturnal rodents studied in the laboratory has led to the formulation of a dual oscillator model in which two circadian clocks, namely a morning (M) and an evening (E) oscillator, are coupled with each other, but also independently entrained by photic cues of dawn and dusk, respectively [37], [38]. As predicted by the dual oscillator model, a seasonal compression and decompression of the daily activity bout is clearly observed in the *Aotus*' activity patterns presented in Figure 1A. For every subject, the two peaks were significantly correlated with the time of sunset and sunrise, respectively (average Pearson's correlation coefficient (range): r_SS_ = 0.89 (0.49−1.00); r_SR_ = 0.70 (0.69−0.99), n = 10 individuals). Although laboratory studies are necessary to determine the involvement of E and M oscillators, our data are consistent with the hypothesis of two oscillators regulating the timing of evening and morning activity peaks.

### Light intensity and masking of activity

**Figure 2:**
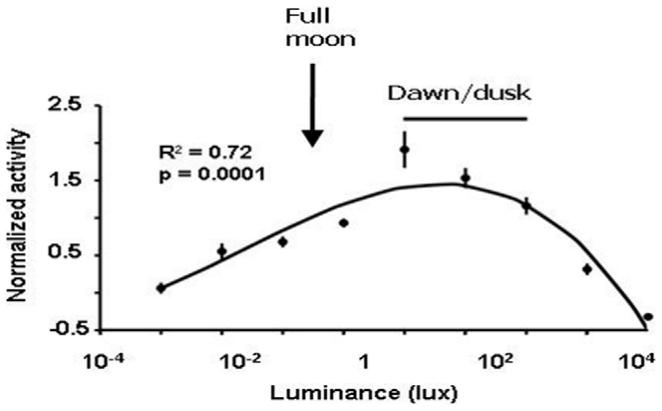
Relationship between locomotor activity levels of *A. azarai* monkeys free-ranging in their natural habitat and luminance levels. Intermediate light intensities positively mask (increase) locomotor activity in *A. azarai*. Each point represents the average normalized activity (± SE) of 9 animals for the range of luminances between one log-unit below and the luminance indicated in the x-axis (for instance, the point corresponding to 10^−2^ lux includes the average activity recorded under luminances >10^−3^ and ≤10^−2^ lux). Luminances corresponding approximately to full-moonlit nights, as well dawn and dusk are indicated. The curve represents a 3^rd^ degree equation best fitted to the points that generated each average shown.

Although crepuscular activity cannot strictly be classified as nocturnal or diurnal, wild *A. azarai* showed higher levels of activity during the night than during the day (Figure 1A, B). Across the year, owl monkeys showed 36.6% (±0.9%) of their total daily activity during the fully dark night hours (2100–0600 h) and 20.3% (±0.9%) during the bright daylight hours (09.00–18:00; Wilcoxon signed-ranked test p = 0.005, negative ranks = 10). This finding is consistent with a true nocturnal phenotype as described for other owl monkeys tested in laboratory conditions [39], [40]. The nocturnal activity was associated with the availability of moonlight and thus might be the output of a circalunar clock, namely an endogenous biological clock with a period close to the lunar cycle of about 24.8 h that is synchronized to the lunar-day. However, studies with other captive owl monkey species have demonstrated that this is unlikely the case [39], [40]. To test whether nocturnal locomotor activity in *A. azarai* in its natural environment may represent a case of positive masking by moonlight, we analyzed the relationship between locomotor activity and ambient luminance as measured in an open savannah area in front of the monkeys' gallery forest habitat. We restricted our analysis to data recorded when ambient temperatures ranged between 15 and 30°C because owl monkeys are rarely active outside this temperature range [32]. Figure 2 shows a striking relationship between locomotor activity and luminance levels (R^2^ = 0.72, p = 0.0001, n = 9 individuals, regression analysis for polynomial third degree equation). The data indicate a 10^−1^–10^3^ lux range of optimal luminance for the expression of locomotor activity. This range corresponds to light intensities typically found during dawn and dusk, as well as during full-moon nights. Thus, our results show that within the temperature range when *A. azarai* is normally active, high locomotor activity was only evident at low to intermediate luminances that are typically found at dawn, dusk or during full-moon nights. These results are consistent with laboratory studies with other owl monkey species that demonstrated that nocturnal activity is the output of a circadian clock synchronized to the 24-h LD cycle, and this activity is highly dependent on the availability of dim light during the dark phase [39], [40].

### Lunar eclipses and inhibition of activity

**Figure 3:**
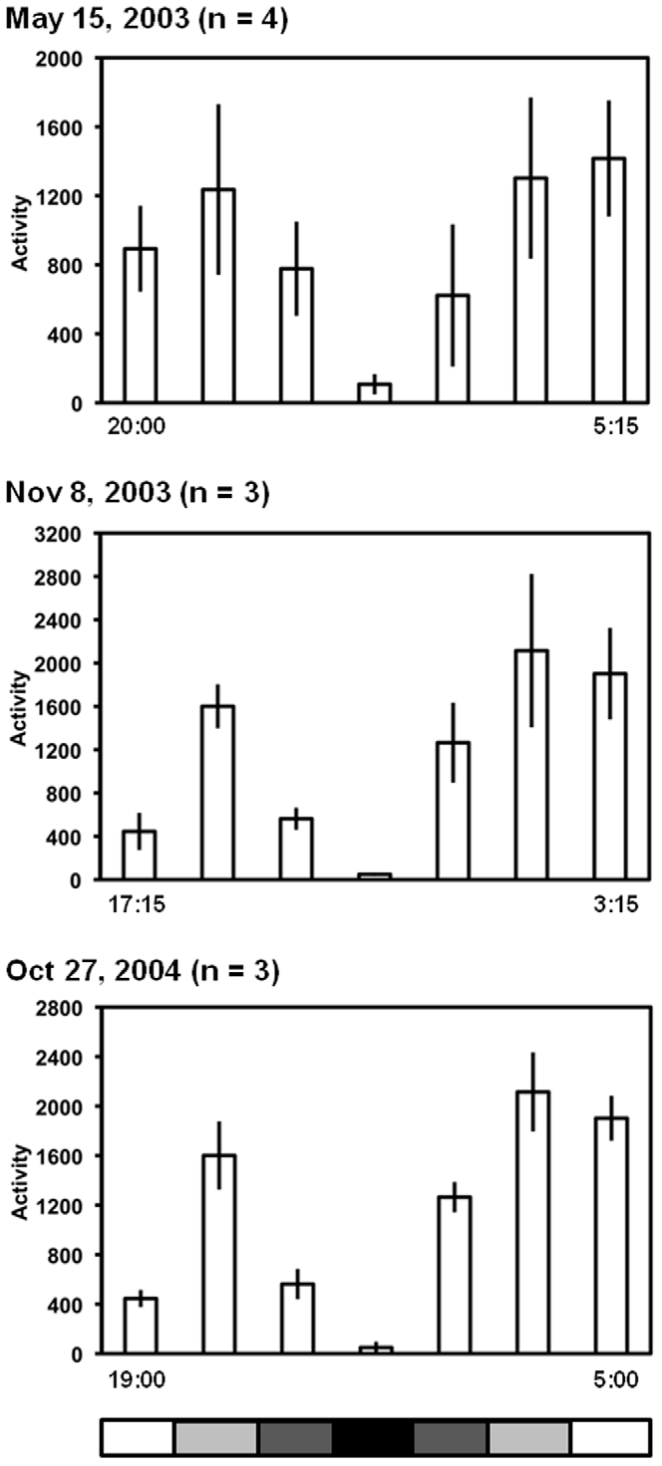
Masking of nocturnal activity by lunar eclipses in wild owl monkeys of the Argentinean Chaco. Activity patterns averaged across individuals for each of the three days when the total lunar eclipses occurred. Averages were calculated for the 2 h before the penumbra phase (left white bar with clock time for the onset of the 2-h window), for the 2-h after the end of the penumbra phase (right white bar with clock time for the offset of the 2-h window), as well as for the penumbra phase (light gray bars), the partial eclipse phase (dark gray bar) and the full eclipse (black bar). The averages represent the mean activity per 5-min interval for the specific phase, regardless of the phase duration. The date of each eclipse is indicated at the top of each graph and the number of recorded subjects is indicated between parentheses. The two hours prior to the eclipses from Nov 8, 2003 and Oct 27, 2004 occurred at times when brighter light intensity likely led to lower levels of activity.

The tight association between locomotor activity and ambient luminance is consistent with the hypothesis of positive masking of circadian locomotor activity by dim light; in other words, activity appears to be inhibited by low and high light intensities, but favored under intermediate ones. Masking is usually tested under laboratory conditions stimulating individuals with light in a dark background, or with darkness in a light background [26], [41]. Although manipulation of light intensity in a natural setting is not possible, three total lunar eclipses that took place during the period of study offered the opportunity to further evaluate the effect of low light intensity on the activity of owl monkeys. Locomotor activity was negatively masked by the absence of light during the lunar eclipses, at times when the animals normally exhibited maximal nocturnal activity (Figure 3). There was an almost complete inhibition of locomotor activity during the full eclipse when moonlight was completely shadowed. Activity was lower during the full eclipse than it was during the partial eclipse, penumbra and before or after the eclipse (Friedman test, X^2^ = 32.35, df = 8, p = 0.000). Low levels of activity have been previously associated with the dim light resulting from lunar eclipses [17]. However, the observational nature of those studies has obvious limitations under the pitch-dark conditions encountered under total lunar eclipses and our results are the first ones showing this association with more reliable and quantitative activity records.

### Ambient temperature and masking of activity

**Figure 4:**
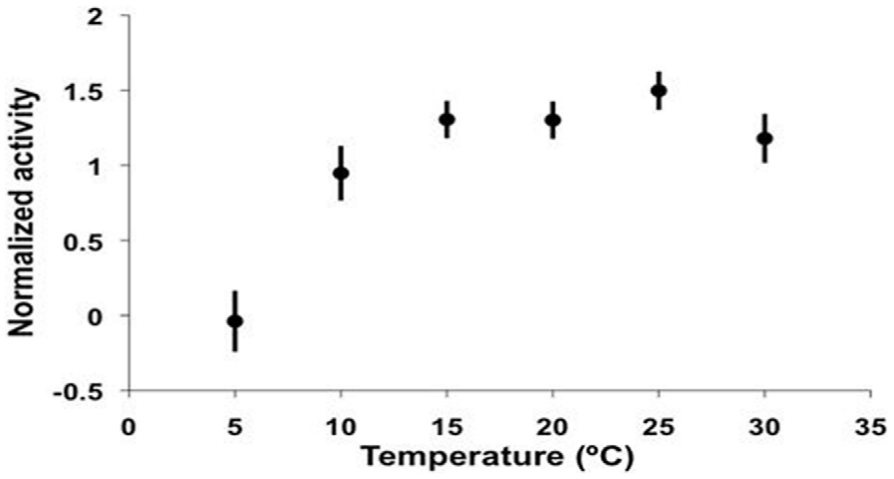
Relationship between locomotor activity levels of *A. azarai* monkeys free-ranging in their natural habitat and ambient temperature. Each point represents the average normalized activity (±SE) of 9 animals for the range of temperatures below the temperature marked in the x-axis. For instance, the point corresponding to 10°C includes the average activities measured between 5 and 10°C. The analysis includes recordings obtained only at optimal luminances for activity, between 10^−1^ and 10^3^ lux (Figure 2).

Low ambient temperatures could be a second environmental factor negatively masking circadian locomotor activity given that nocturnal activity, even during nights of full moon, was diminished during the winter months (Figure 1A). To test this prediction, we examined the relationship between activity level and ambient temperature during optimal luminance conditions (10^−1^–10^3^ lux, Figure 2). Even under optimal luminance conditions, activity tended to be maximum between 15–25°C, reduced when temperatures were slightly lower or higher and almost non-existent when 5°C or lower (Figure 4, one-way ANOVA with temperature as main factor: F_1,46_ = 24.49, p = 0.000).

Our results represent the first long-term field study providing direct evidence for environmental masking in the only nocturnal anthropoid primate. These data indicate that although rhythmic locomotor activity may represent the output of a circadian clock, nocturnality, namely the relative predominance of locomotor activity during the dark phase of the natural LD cycle, is the result of fine-tuned masking of circadian rhythmicity by environmental light and temperature. The behavioral outcome of this masking is nocturnal activity that is maximal during relatively warm, moonlit nights. Whereas laboratory studies have pointed to the importance of masking in determining environmental factors causing switches from nocturnal activity patterns to diurnal ones or vice versa [27], [28], [42], our study underscores the importance of masking in determining the daily activity patterns of animals living in the wild. It also suggests that moonlight has probably selected for positive masking by dim light as a key adaptation for the exploitation of the nocturnal niche by primates [8], [11].

It is still a matter of controversy whether ancestral primates were nocturnal, diurnal or had patterns of activity that involved activity during both night and day [3], [43]. Equally controversial is how frequent transitions between diurnality and nocturnality, and vice versa, occurred throughout primate evolution [44]. The present study indicates that modifications in sensory systems, that relay information on environmental masking factors to effector systems which sustain locomotor activity, can influence those evolutionary changes. The data also highlight the importance of placing any analysis of the evolution of primate opsins [43], [45], [46] in the context of positive masking of locomotor activity by nocturnal moonlight. Our results clearly indicate that the masking effects that ambient luminance and temperature exert on locomotor activity have been selected as key proximate mechanisms to shape the temporal niche of owl monkeys within a gradient between nocturnality and diurnality.

## Materials and Methods

### Ethics Statement

The capturing and immobilization of individuals for the fitting of the actometer collars was done in general agreement with established protocols by the Institutional Animal Care and Use Committees of the Zoological Society of San Diego (#146) and the University of Pennsylvania (#801089). In accordance with Argentinean regulations, both the National Wildlife Directorate and the provincial Wildlife Department were at all times informed of procedures. All procedures were classified as Category B indicating that although there was potential for pain/distress, relief was provided by analgesics/anesthetics/sedatives as appropriate.

Animals were fitted with actimeter collars (Actiwatch AW4) as previously described [7], [29]. Actimeters were programmed to record accumulated activity counts every 5 min. For Figure 1, activity was normalized by subtracting the mean of all 5-min values for each actimeter data file from each individual value and dividing the resulting number by the standard deviation of that mean. 15-min average activity was calculated to construct the actograms and wave profiles. For correlations with luminance and temperature, 1-h average activity was calculated for 9 animals because no luminance data were available for the 10^th^ animal. The hourly means were normalized like the 15-min ones.

The normalized hourly means were then averaged or added to characterize diurnal or nocturnal activity. For all months, diurnal activity was defined as activity occurring during the fully bright part of the 24-h day (0900–1800hs), whereas nocturnal activity was considered to take place during the completely dark part of the 24-h day (2100–0600hs). Dawn and dusk were excluded from the definition of nocturnal/diurnal activity because crepuscular activity can be considered neither nocturnal nor diurnal. This method leads to a clearer distinction between activity that takes place truly during the night or truly during the day.

Luminance was monitored every 5 min with an Actiwatch-LP actimeter/luxmeter data logger device with remote photocell (Cambridge Neurotechnology, UK), placed in a small water tight transparent acrylic box fixed on top of a 2-m high post situated in an open area contiguous to the study site with the photocell directed upwards to the zenith. This device can automatically record light measurements in the 0.01–65000 Lux range and store the data collected for approximately 30 days. The luminance levels perceived by the monkeys were probably 1–2 log units lower than the measured ones, because the luxmeter was placed outside the forest. Temperature was recorded hourly with a Stowaway XTI data logger placed at the study site. For data presented in Figure 2, normalized hourly activity was ranked according to environmental temperature and only data sampled between 15 and 30°C was included in the analysis. Temperatures above 30°C are only encountered during the early afternoon when the monkeys are notoriously inactive [32]. For data presented in Figure 3, we first obtained the individual average amount of activity for each of the periods considered, then computed an across individual average. For Figure 4, normalized hourly activity was ranked according to luminance and only data sampled at luminances between 10^−1^–10^3^ lux were included in the analysis. The measures of central tendency and dispersion are arithmetic means ± s.e.m. Astronomical data were obtained from The Astronomical Almanac (aa.usno.navy.mil/data) and the NASA Eclipse Website (http://eclipse.gsfc.nasa.gov/eclipse.html).
